# Strategies To Assess Hypoxic/HIF-1-Active Cancer Cells for the Development of Innovative Radiation Therapy

**DOI:** 10.3390/cancers3033610

**Published:** 2011-09-15

**Authors:** Chan Joo Yeom, Lihua Zeng, Yuxi Zhu, Masahiro Hiraoka, Hiroshi Harada

**Affiliations:** 1 Group of Radiation and Tumor Biology, Career-Path Promotion Unit for Young Life Scientists, Kyoto University, Yoshida Konoe-cho, Sakyo-ku, Kyoto 606-8501, Japan; E-Mails: cjyeom@kuhp.kyoto-u.ac.jp (C.J.Y.); lzwei1998@gmail.com (L.Z.); zhuyuxi@kuhp.kyoto-u.ac.jp (Y.Z.); 2 Department of Radiation Oncology and Image-applied Therapy, Kyoto University Graduate School of Medicine, 54 Shogoin Kawahara-cho, Sakyo-ku, Kyoto 606-8507, Japan; E-Mail: hiraok@kuhp.kyoto-u.ac.jp

**Keywords:** radiation therapy, tumor hypoxia, radioresistance, hypoxia-inducible factor 1 (HIF-1), molecular imaging

## Abstract

Local tumor recurrence and distant tumor metastasis frequently occur after radiation therapy and result in the death of cancer patients. These problems are caused, at least in part, by a tumor-specific oxygen-poor microenvironment, hypoxia. Oxygen-deprivation is known to inhibit the chemical ionization of both intracellular macro-molecules and water, *etc.*, and thus reduce the cytotoxic effects of radiation. Moreover, DNA damage produced by free radicals is known to be more repairable under hypoxia than normoxia. Hypoxia is also known to induce biological tumor radioresistance through the activation of a transcription factor, hypoxia-inducible factor 1 (HIF-1). Several potential strategies have been devised in radiation therapy to overcome these problems; however, they have not yet achieved a complete remission. It is essential to reveal the intratumoral localization and dynamics of hypoxic/HIF-1-active tumor cells during tumor growth and after radiation therapy, then exploit the information to develop innovative therapeutic strategies, and finally damage radioresistant cells. In this review, we overview problems caused by hypoxia/HIF-1-active cells in radiation therapy for cancer and introduce strategies to assess intratumoral hypoxia/HIF-1 activity.

## 1. Introduction

Chemotherapy, radiation therapy, and combinations thereof are nowadays playing important roles in cancer therapy; however, even the most innovative strategies have failed to achieve a complete remission, and patients often suffer from local tumor recurrence and/or distant metastases. This problem is, at least in part, caused by the chemo- and/or radio-resistance of cancer cells in most malignant tumors. Whether individual cancer cells are resistant to chemo- and or radiotherapy is known to be influenced by various intrinsic and extrinsic factors. Evidence accumulated through extensive basic and clinical research has suggested that one of the most influential of these factors is hypoxia, the low oxygen condition seen in most solid tumors [1-4].

Because of the typical characteristics of cancer cells, such as aberrantly accelerated proliferation and high metabolic demands, the “oxygen demand in cancer cells” greatly exceeds the “oxygen-supply to them”, causing hypoxic regions in most malignant solid tumors [3-5]. Tumor blood vessels are functionally defective, which is also a causative factor of hypoxic regions [6]. Depletion of oxygen directly disturbs radiation-induced production of reactive and cytotoxic species [2,7]. Moreover, hypoxia induces tumor radioresistance through the activation of a transcription factor, hypoxia-inducible factor 1 (HIF-1) [8-14]. Thus, cancer cells better survive radiation under hypoxic conditions. Hypoxic tumor cells are known to survive conventional chemotherapies, too [15], because they exist far from tumor blood vessels and therefore are not delivered effective doses of anti-cancer drugs. HIF-1 seems to function in chemoresistance as well as radioresistance; the expression of a multi-drug resistance gene is under the control of HIF-1 [16].

Several strategies have been developed to overcome these problems. Fractionated radiation therapy aims to efficiently kill hypoxic tumor cells by repeatedly delivering radiation to a malignant tumor in which hypoxic cells have been reoxygenated as a result of ex-irradiation [17-19]. Hypoxia image-guided radiation therapy (Hypo-IGRT) aims to deliver a booster dose of radiation especially to small target fractions which are detected in a malignant tumor through imaging strategies for tumor hypoxia [20]. Hypoxia-selective cytotoxins/drugs act to directly damage hypoxic tumor cells [7]. HIF-1 inhibitors act to suppress HIF-1-mediated tumor radioresistance [10,21].

My colleagues and I have performed basic research using tumor-bearing mice to analyze the spatio-temporal dynamics of intratumoral hypoxia and HIF-1 activity. We have revealed that the location of hypoxic tumor cells/HIF-1 activity changes dramatically as a tumor grows [22,23]. Immunohistochemical analysis combined with optical real-time imaging for intratumoral HIF-1 activity revealed that ionizing radiation dramatically alters the distribution of oxygen and nutrients in a solid tumor, triggering a transient decrease and subsequent increase in intratumoral HIF-1 activity [9,24,25]. Moreover, when we administered a HIF-1 inhibitor to tumor-bearing mice and suppressed the radiation-induced activation of HIF-1, we could enhance the therapeutic effect of radiation [9]. On the other hand, the administration of a HIF-1 inhibitor at the wrong time can suppress rather than enhance the effect of radiation therapy because its anti-angiogenic effect increases the radioresistant hypoxic fraction [9]. All of these results highlight the importance of assessing the localization and dynamics of hypoxia/HIF-1 activity during the growth of human cancers and after radiation therapy. Then, it is critical to optimize the treatment protocols of innovative strategies [8].

Several methods have been developed to assess hypoxia and HIF-1 activity in cancers. Oxygen-sensitive electrodes [26-28], phosphorescence imaging [29,30], and immunohistochemical staining using intrinsic and extrinsic hypoxic markers are well established. They are useful not only for animal, but also human tumors, but there are some limitations; these methods are highly invasive and sometimes suffer from selection bias. Noninvasive molecular imaging techniques using optical, nuclear medicine, and magnetic resonance (MR) imaging are alternative approaches. In this review, we overview the problems caused by hypoxia/HIF-1-active cells in cancer therapy and introduce potential new strategies to assess hypoxia and/or HIF-1-active cells in malignant solid tumors.

## 2. Hypoxia and HIF-1

### 2.1. Tumor Hypoxia

The microenvironment of malignant solid tumors is totally different from that of normal tissues, being characterized by extreme diversities in ionic strength, pH, the distribution of nutrients, and oxygen concentrations [3,4,8,15]. The heterogeneity of intratumoral oxygen concentrations in particular has drawn considerable attention in both cancer research and cancer therapy since Thomlinson and Gray proposed the existence of hypoxic regions in solid tumors and its relevance to tumor radioresistance in 1955 [3].

**Figure 1.**
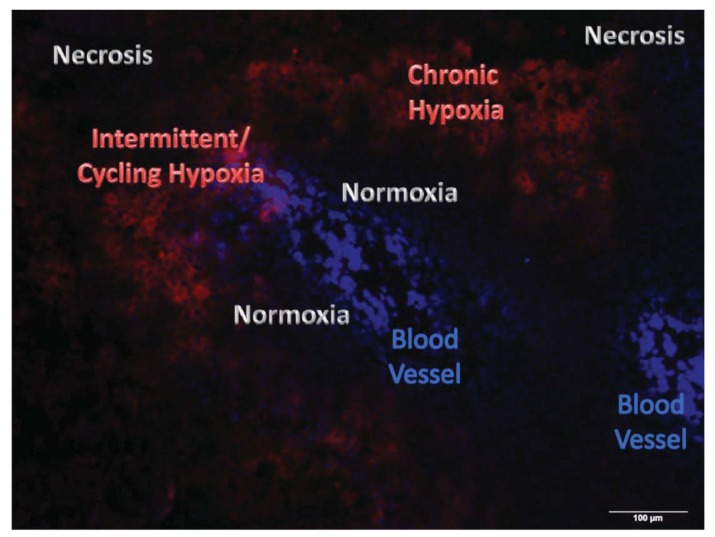
Spatial relationship between tumor blood vessels and hypoxic regions in a malignant solid tumor. A tumor-bearing mouse with human cervical cancer cells, HeLa, was administrated with a hypoxia-marker, pimonidazole (red), and a perfusion marker, Hoechst33342 (blue), 90 and 1 min before sacrificing the animal, respectively. The tumor xenograft was surgically excised and its frozen section was stained with anti-pimonidazole antibody. Chronic hypoxia exists 70–100 μm from tumor blood vessels. Intermittent/cycling hypoxia influenced by fluctuations in tumor blood flow can be detected proximal to tumor blood vessels.

Tumor hypoxia can be categorized as “chronic” and “acute” according to the causative factors and the duration in which cancer cells are exposed to hypoxic conditions [8]. Cancerous cells commonly possess characteristics such as deregulated cellular energetics, sustained proliferative signaling, evasion of growth suppressors, and replicative immortality [5]. In most malignant solid tumors the vasculature is functionally and structurally defective [6]. These characteristics lead to an imbalance between oxygen supply to and oxygen consumption in a malignant solid tumor, and can cause a highly heterogeneous and severely compromised oxygenation of tumors [3,4,8,15]. Tumor cells proliferate and grow actively only when supplied with oxygen and nutrients; therefore, most malignant tumors individually grow as a conglomerate of so-called “micro tumor cords”. A tumor blood vessel is surrounded by actively proliferating cancer cells (normoxic regions) [3,15,31]. On the other hand, cancer cells inevitably die in areas approximately 100 μm from tumor blood vessels (necrotic regions) [3,15,31]. Between these regions, there exist so-called chronic hypoxic areas, in which cancer cells obtain very minimal levels of oxygen, enough for their survival, but not for their active proliferation (Figure 1) [3,15,31].

Recently, acute/intermittent/cycling hypoxia has also received much attention because of its relevance to the malignancy and radioresistance of cancer cells. Acute hypoxia was first recognized by Brown *et al.* in 1979 [32], who mentioned that a malformed tumor vasculature causes the transient opening and closing of blood vessels, changes in the blood flow rate, fluctuations in perfusion, and ultimately the generation of a transient hypoxia. Because of these causative factors, acute hypoxia can appear even within 70 μm of tumor blood vessels (Figure 1). Subsequent studies showed that at least 20% of cancer cells experience acute hypoxia in malignant solid tumors [33,34].

Clinical studies using a computerized polarographic needle electrode revealed that, in malignant tumors, such as uterine cervix cancers, head and neck cancers, and breast cancers, overall median partial oxygen pressure (pO_2_) is about 10 mm Hg and the overall hypoxic fraction (pO_2_ ≤ 2.5 mm Hg) is approximately 25% [35]. In contrast, no pO_2_ values lower than 12.5 mm Hg were found in normal tissues, such as normal breast tissues [36].

### 2.2. Treatment Failure and Increase in a Wide Range of Tumor Malignancies Caused by Hypoxia

#### 2.2.1. Radioresistance

The radioresistance of cancer cells is known to be influenced by various extrinsic as well as intrinsic factors. Hypoxia is one of the most influential factors [1-4]. Ionizing radiation causes ionization in or close to the genomic DNA of target cancer cells, and produces radicals [7]. The DNA radicals can be oxidized in the presence of oxygen, keeping the damage unrepairable. Meanwhile, in the absence of oxygen, the DNA radicals are reduced by compounds containing sulfhydryl groups (SH groups), which restore the DNA to its original form. Therefore, DNA damage, especially irreparable double stranded breaks, is significantly less severe in the absence of oxygen molecules. In addition to such a mechanism, it has also been reported that depletion of oxygen directly disturbs radiation-induced production of reactive and cytotoxic species [2,7].

Hypoxia-mediated radioresistance is attributed to biological as well as chemical mechanisms. Hypoxic stimuli trigger changes in the activities of both the “DNA damage repair pathway” [37] and the “cell death/survival signaling pathway”. Moreover, recent advances in molecular and cellular biology revealed that a transcription factor, hypoxia-inducible factor 1 (HIF-1), plays a pivotal role in tumor radioresistance (see Section 2.3. for details) [8].

Consistent with these notions, clonogenic survival assays have showed that cancer cells become 2–3 times more radioresistant under hypoxic conditions than normoxic conditions [7]. Also, there is accumulated clinical evidence that the size of the intratumoral hypoxic fraction correlates well with the poor prognosis of cancer patients after radiation therapy [7,38].

#### 2.2.2. Chemoresistance

Multiple mechanisms function in the chemoresistance of cancer cells in hypoxic regions of locally advanced solid tumors [15,39]. First, because hypoxic regions occur far from functional vasculatures, the diffusion and delivery of most anticancer drugs are not extensive enough to show a cytotoxic effect [40-42]. Second, conventional anti-cancer drugs, such as alkylating agents and antimetabolites, are known to be less effective under hypoxic conditions. Because these kinds of drugs can effectively kill highly proliferating cancer cells, hypoxic tumor cells, which are less proliferating and sometimes even dormant, can tolerate them [43]. Third, the cytotoxicity of some anticancer drugs is known to depend on molecular oxygen. For example, bleomycin is reported to produce a pseudoenzyme that reacts with oxygen and generates both superoxide and hydroxide free radicals, and consequently, cleaves the genomic DNA of target cancer cells. Therefore, its cytotoxic effect dramatically decreases under low O_2_ conditions [44,45]. Fourth, hypoxia upregulates the expression of genes involved in drug resistance, such as p-glycoprotein which is responsible for the export of anti-cancer drugs from inside to outside of cancer cells [16,46]. Finally, there is evidence that hypoxia can enhance genetic instability in tumor cells, thus allowing a more rapid development of drug resistance [47].

#### 2.2.3. Metastasis and Angiogenesis

In addition to mediating resistance to conventional treatments, hypoxia is known to increase the metastatic and angiogenic potential of cancer cells. Cancer patients with relatively more hypoxic regions have a tendency to suffer from distant metastasis as well as local recurrence regardless of whether the initial treatment is surgery or radiation therapy [48]. Recent molecular biological analyses have revealed that hypoxia stimulates the expression of a number of genes involved in metastatic cascades, such as the gene for lysyl oxidase, the chemokine receptor CXCR4, and osteopoetin [49-51]. In addition, cancer cells under hypoxic conditions trigger angiogenesis in order to improve surrounding conditions and obtain enough oxygen and nutrients for their survival [52].

### 2.3. Treatment Failure Caused by HIF-1

By activating a transcription factor hypoxia-inducible factor 1 (HIF-1), cancer cells induce the expression of various genes responsible for not only the “adaptation of cellular metabolism to hypoxia (switch from oxidative to anoxic respiration) [53]”, “escape from hypoxia (invasion and metastasis of cancer cells) [49,54]”, and “improvement of severe hypoxic conditions (angiogenesis) [55,56]” but also “resistance to chemotherapy and radiation therapy”.

#### 2.3.1. Regulation of HIF-1 Expression and Activity

**Figure 2.**
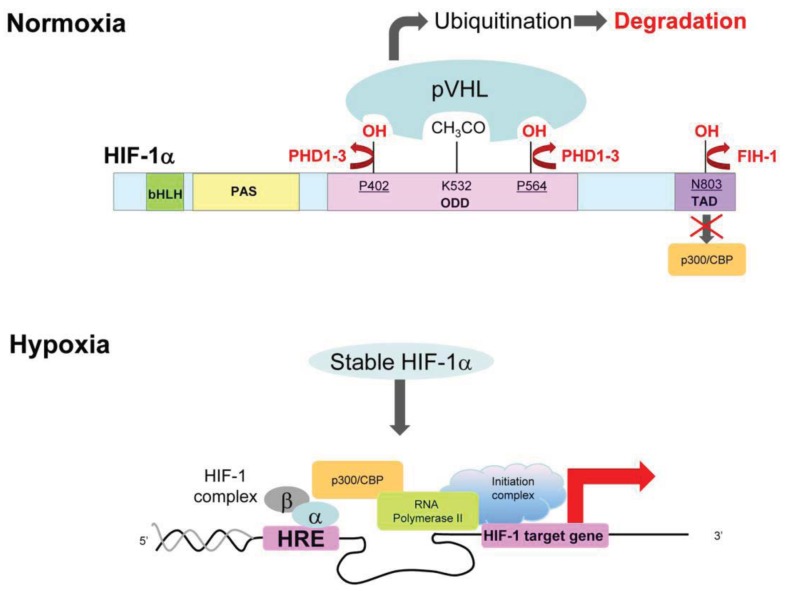
Hypoxia-dependent regulation of HIF-1 activity.

HIF-1 is a heterodimeric transcription factor composed of alpha (HIF-1α) and beta (HIF-1β/ARNT) subunits [57]. Its hypoxia-dependent activity is mainly regulated through the post-translational modification of the HIF-1α subunit (Figure 2).

The best-characterized regulatory mechanism is that modulating HIF-1α's stability. In the presence of oxygen, prolyl hydroxylation and subsequent ubiquitination of the oxygen-dependent degradation (ODD) domain of HIF-1α by prolyl hydroxylases (PHDs) and von-Hippel Lindau (VHL)-containing E3 ubiquitin-protein ligase, respectively, leads to the rapid degradation of the HIF-1α protein [58-62]. On the other hand, in the absence of oxygen, HIF-1α becomes stable because oxygen-depletion directly suppresses the activity of PHDs [60]. The stabilized HIF-1α interacts with its binding partner, HIF-1β, and forms a heterodimer, HIF-1 [57]. HIF-1 binds to its cognate DNA sequence, the hypoxic-responsive element (HRE), and induces the expression of various genes related to angiogenesis, metastasis, glycolysis, chemo/radioresistance and so on [49-51,55,63].

In addition to the regulation of HIF-1α's stability, another post-translational modification of HIF-1α functions in the regulation of the transactivation activity of HIF-1. Under normoxic conditions, factor inhibiting HIF-1 (FIH-1) becomes active and hydroxylates an asparagine residue (N803) of HIF-1α [59,62,64]. The asparaginyl hydroxylation blocks the interaction of HIF-1α with the transcriptional co-factor p300 and CBP, resulting in the suppression of HIF-1's transactivation activity. Because oxygen is a substrate of FIH-1, HIF-1 's transactivation activity can be restored under hypoxic conditions.

#### 2.3.2. Function of HIF-1 in Radioresistance and Chemoresistance of Cancer Cells

Through preclinical studies using a pharmacological HIF-1 inhibitor, YC-1, a dominant negative mutant of HIF-1α, or short hairpin/short interfering RNA against HIF-1α, it has been extensively confirmed that inhibition of intratumoral HIF-1 activity delayed tumor growth after radiation therapy [9-11,65,66]. In clinical studies, it has been repeatedly confirmed that HIF-1α expression correlates with a poor prognosis for various cancer patients after radiation therapy [67,68]. All of these results imply that HIF-1 has a certain biological function to induce a radioresistant phenotype of cancer cells. Actually, HIF-1-mediated radioresistance has been revealed recently. Namely: (1) radiation activates HIF-1 in a solid tumor; (2) HIF-1 induces the expression of VEGF; (3) VEGF protects endothelial cells from the cytotoxic effects of radiation, and (4) the radio-protected tumor blood vessels assure the supply of oxygen and nutrients to tumour cells and promote tumour growth [10,14,24]. As for the chemoresistance of cancer cells, HIF-1 is known to play an important role in the hypoxia-dependent expression of p-glycoprotein, as mentioned in Section 2.2.2. [16,46].

#### 2.3.3. Function of HIF-1 in Angiogenesis, Metabolic Reprogramming, Invasion and Metastasis

HIF-1 plays pivotal roles in angiogenesis, metabolic reprogramming, and invasion & metastasis for the improvement of, adaptation to, and evasion from hypoxic conditions, respectively [49,50,55,69-71]. Upregulation of HIF-1 activity caused by intratumoral hypoxia is involved in the induction of vascular endothelial growth factor (VEGF), which is a glycoprotein responsible for angiogenesis and vasculogenesis [55]. In addition, HIF-1 induces the expression of genes encoding glucose transporters, glycolytic enzymes and lactate dehydrogenase *etc.*, all of which function in glycolysis and lactic acid fermentation [53,72]. At the same time, HIF-1-dependent genes decrease both mitochondrial metabolism [73] and mitochondrial mass [71,74] leading to efficient production of ATP even under oxygen-deprived conditions through anaerobic respiration but not through oxidative respiration driven by the tricarboxylic acid (TCA) cycle and electron transport chain (ETC). Such reprogramming also functions in the decrease in the level of cytotoxic reactive oxygen species (ROS) produced through incomplete oxidative phosphorylation under hypoxic conditions [53,69,73]. Furthermore, HIF-1 is known to trigger the metastasis of cancer cells under hypoxic conditions by including epithelial-mesenchymal transition (EMT) and expression of the Met protooncogene and lysyl oxidase [50,75].

## 3. Direct Measurement of Low Oxygen Conditions

Several methods have been devised and developed to assess hypoxia and HIF-1 activity in cancers. In this section, we introduce several methods to detect low oxygen conditions in malignant solid tumors (Table 1).

### 3.1. Polarographic Needle Electrode

In 1986, Weiss and Felckenstein pioneered the use of a polarographic needle electrode for measuring the partial pressure of oxygen (pO_2_) in malignant tumors. The pO_2_ in tumors could be directly quantified without any artifacts caused by compression [28]. By using this technique, the existence of hypoxia in solid tumors was significantly described by Vaupel *et al.* [36]. In malignant tumors, such as uterine cervix cancer, head and neck cancer, and breast cancer, overall median pO_2_ is about 10 mm Hg and the overall hypoxic fraction (pO_2_ ≤ 2.5 mm Hg) is approximately 25%. In contrast, pO_2_ values lower than 12.5 mm Hg were not found in normal tissues, such as normal breast tissue. This technique has several disadvantages for clinical application including tissue damage, the need for great expertise, and limitations to accessible tissue sampling. Moreover, although the polarographic needle electrode has already been computerized, there remains a possibility that it causes sampling error and leads to artificial and biased data.

### 3.2. Blood Oxygen Level-Dependent Contrast Magnetic Resonance Imaging (BOLD-MRI)

BOLD-MRI is a noninvasive imaging technique reflecting the changes in blood oxygenation based on distinguishing paramagnetic deoxyhemoglobin as an endogenous contrast agent. In the 1990s, Prasad *et al.* pioneered the application of BOLD-MRI to the non-invasive observation of renal oxygenation [76]. They reported that BOLD-MRI is so sensitive that it can monitor renal hypoxia which cannot be detected using a well-known hypoxia marker, pimonidazole, through immunohistochemistry [77]. Padhani *et al.* applied BOLD-MRI to the detection of tumor hypoxia [78]. Notwithstanding the fact that BOLD-MRI provides noninvasive information about blood oxygenation levels with high spatiotemporal resolution and high sensitivity, it is not clear if blood oxygenation levels are directly reflected in tumor tissue oxygenation. Moreover, this technique is not quantitative and can be easily influenced by many physical factors including flow effects, pH and temperature [79], preventing its routine clinical use.

### 3.3. Dynamic Contrast Enhanced Magnetic Resonance Imaging (DCE-MRI)

DCE-MRI is a non-invasive method used to evaluate regional tumor blood flow as the temporal distribution of a small-molecular-weight tracer, D_2_O [80]. Preclinical and clinical studies have suggested that DCE-MRI provides valid information about the oxygen tension and localization of hypoxic regions in a solid tumor [81-83]. Some researchers have demonstrated the feasibility of integrating the DCE-MRI technique into intensity-modulated radiotherapy (IMRT) in order to define a biological target volume (BTV) for advanced dose painting [84].

### 3.4. ^19^F magnetic Resonance Imaging (^19^F-MRI)

^19^F-MRI relaxometric mapping was established by Magat *et al.* to analyze the spontaneous fluctuations of pO_2_ over time in tumor xenografts [85]. After the intratumoral injection of a fluorine compound, hexafluorobenzene (HFB), the relaxation rate (1/T1) correlates linearly with the dissolved oxygen concentration. To acquire parametric images of the T1 relaxation time with a high spatial and temporal resolution, they used a SNAP inversion-recovery sequence at 4.7 T. Although ^19^F MRI is appropriate for detecting rapid changes in tumor oxygenation, the measurements are subject to flow artifacts and several conditions including temperature, dilution, pH, common proteins, and blood can easily affect the sensitivity of some ^19^F-MRI compounds [86].

### 3.5. Electron Paramagnetic Resonance (EPR) Imaging

Dynamic three-dimensional electron paramagnetic resonance imaging (EPRI) is a recently developed method [87,88]. EPRI is a low-field magnetic resonance technique that accomplishes the three-dimensional and quantitative evaluation of oxygenation status with a 1-2-mm spatial resolution every 2–3 min. The real-time imaging of tissue hypoxia can be quantitatively accomplished by the collisional interaction of an exogenously administered paramagnetic tracer with molecular oxygen.

### 3.6. Positron Emission Tomography (PET) Imaging

Recently, the research field for molecular imaging using PET has been growing rapidly because of the development of several small-animal PET high-resolution cameras and fused imaging modalities, such as PET/CT and PET/MRI which provide not only functional but also detailed anatomical information [89]. For imaging with the PET system, ^18^F, ^124^I, and ^60/64^Cu are commonly used as positron-emitting radioisotopes as they can be labeled with organic molecular markers for hypoxia. Several different molecular markers have been tested as hypoxic tracers for PET imaging, such as nitromidazoles and bis(thiosemicarbazones).

#### 3.6.1. Radiopharmaceutical Characteristics of Nitroimidazoles

Analogues of 2-nitromidazole, such as fluoromisonidazole (FMISO), fluoroetanidazole (FETA), fluoroerythronitroimidazole (FETNIM), fluoroazomycinarabinofuranoside (FAZA), and EF5, can be used as hypoxic markers for PET imaging. The mechanism of action of the 2-nitroimidazole derivatives is well understood; they can be activated through reduction and retained through covalent binding to thiol groups of arbitrary polypeptides and proteins in hypoxic cells. On the other hand, in the presence of oxygen the reductively activated tracer of a 2-nitromidazole analogue returns to its original form by reacting with O_2_. In the 1970s and 1980s, many researchers intensively exploited the characteristics of nitroimidazole analogues as oxygen mimetics to increase cytotoxic effects of ionizing radiation toward hypoxic cells. Varghese *et al.* demonstrated in 1976 that ^14^C-labeled misonidazole formed adducts in hypoxic cells *in vitro* and *in vivo* [90]. The potential use of radio-labeled nitroimidazoles for imaging hypoxia was suggested by Chapman [91]. He and others demonstrated that ^14^C-labeled derivatives of *N*-alkyl-2-nitroimidazoles were reduced to their active form and trapped in living hypoxic cells not in necrotic regions of tumors [92-94].

#### 3.6.2. The First-Generation Nitroimidazole Markers

^18^F-labeled misonidazole (^18^F-FMISO) was developed as a first-generation nitromidazole marker. ^18^F-FMISO PET has been widely used over 15 years and made significant contributions to research [95]. This radiotracer can identify the heterogeneous distribution of hypoxic regions in human tumors, such as brain tumors [96,97]. The lack of a correlation between hypoxia and glucose metabolism was revealed in a non-small cell lung cancer by PET imaging with ^18^F-FMISO and ^18^F-FDG [98]. Moreover, the prognostic effect of ^18^F-FMISO PET on survival in head and neck cancer was stronger than that of ^18^F-FDG [99]. Although ^18^F-FMISO has been extensively evaluated as a standard for PET imaging in preclinical and clinical studies, it has been criticized for its slow body clearance because of the partitioning mechanism and poor hypoxia to background ratios. Notably, ^18^F-FMISO was not suitable for the detection of hypoxia in variant soft tissue tumors [100]. To overcome these problems, various second-generation nitromidazole hypoxic markers, such as ^18^F-flouroetanidazole (^18^F-FETA), fluoroerythronitroimidazole (FETNIM), and EF5, have been developed.

#### 3.6.3. The Second-Generation Nitroimidazole Markers

^18^F-flouroetanidazole (^18^F-FETA) is a fluorinated derivative of etanidazole, introduced by Rasey *et al.* as a promising new agent for hypoxia imaging [101]. According to their biodistribution results, the retention of ^18^F-FETA in liver and lung was significantly lower than that of ^18^F-FMISO in mice. Fewer ^18^F-FETA metabolites were found in plasma and urine, even though the oxygen-dependent binding of the two tracers was very similar. ^18^F-fluoroerythronitroimidazole (^18^F-FETNIM) has been also developed as a hypoxic marker. The uptake of both ^18^F-FETNIM and ^18^F-FMISO correlated with oxygenation status in C3H mammary carcinomas [102]. ^18^F-fluoroazomycinarabinofuranoside (^18^F-FAZA) is a more hydrophilic derivative and therefore has a faster clearance than ^18^F-FMISO [103]. ^18^F-2-2(nitro-1[*H*-imidazol-1-yl)-*N*-(2,2,3,3,3-pentafluoropropyl)-acetamide (^18^F-EF5) is another promising tracer of tumor hypoxia and EF5 was reported to be the most stable 2-nitromidazole derivative [104]. Recently, a human study of this tracer has been conducted to examine whether it functions as a prognostic hypoxia marker [105]. A problem in these kinds of imaging probes (especially with the EF5) is the difficulties in their synthesis and radio-labeling with ^18^F.

#### 3.6.4. Bis(thiosemicarbazone) Compounds

A copper-containing bis(thiosemicarbazone) complex, Cu(II)-diacetyl-bis (*N*^4^-methylthiosemi-carbazone, Cu-ATSM), labeled with a positron emitting isotope of copper (^60^Cu, ^61^Cu, ^62^Cu or ^64^Cu) has been developed as an alternative to ^18^F-FMISO based on the bioreductive character of the metal contained in the copper-complex exposed to oxygen depletion. It has been known that the complex of DTS with Cu(II) has antitumor properties. Fujibayashi *et al.* first evaluated the feasibility of Cu-ATSM as a hypoxia imaging agent using an ischemic rat heart model in 1997 [106]. Compared to ^18^F-FMISO, Cu-ATSM is taken up more rapidly and has a higher hypoxic-to-normoxic ratio [107, 08]. The validity of Cu-ATSM as a hypoxia imaging agent has been demonstrated in animal [107-109] and human [110-112] studies.

## 4. Measuring Exogenous and Endogenous Hypoxia Markers

In addition to the above-mentioned methods to directly assess heterogeneous oxygen distribution in malignant solid tumors, immunohistochemical approaches and optical imaging have also contributed to basic cancer research and clinical activities (Table 2).

### 4.1. Immunohistochemistry

As mentioned, nitroimidazole derivatives are specifically reduced under hypoxic conditions and form a covalent bond with thiol groups of arbitrary proteins in cells. Using the characteristics of nitroimidazole derivatives, tumor hypoxia can be detected by immunohistochemical analyses as well as PET imaging. First, cancer patients should be administered with a nitroimidazole derivative, e.g., pimonidazole and EF5 [113,114], and then, the cancer can be surgically excised and subjected to immunostaining with anti-pimonidazole or anti-EF5 antibody, respectively.

In addition to such a strategy, it is important to assess HIF-1 activity because of its pivotal role in malignant phenotypes and chemo/radioresistance of cancer cells (see Section 2.3 for details). Because HIF-1 activity is mainly dependent on the stability of the HIF-1α protein, we can indirectly but quite accurately monitor the intratumoral localization of HIF-1-active cells through immunostaining with anti-HIF-1α antibody. Downstream target genes of HIF-1 are also useful for this purpose as intrinsic markers. Notably, the expression levels of glucose transporter-1 (GLUT-1) and/or carbonic anhydrase IX (CAIX) were proved to correlate with the intensity of pimonidazole staining, a poor prognosis in patients with deep, large, high-grade soft tissue sarcomas [115], resistance of head and neck cancers to platinum-based radio-chemotherapy [116], and the frequency of local recurrence of glottic squamous cell carcinoma [117], *etc*.

An advantage of the immunohistochemical approach is that one can obtain histological and morphological information about the localization of low pO_2_ and/or HIF-1-positive regions at the micro level. On the other hand, it has some disadvantages: it is highly invasive, and moreover, one cannot follow-up the dynamics and changes in hypoxia in the same tumor sequentially.

### 4.2. Reporter Gene Imaging

#### 4.2.1. Development of HIF-1-Dependent Reporter Genes

Imaging using a HIF-1-dependent promoter is the only strategy which enables us to assess intratumoral HIF-1 activity. Various HREs, such as murin phosphoglycerate kinase-1 (PGK-1) HRE, human enolase (ENO) HRE, murine lactate dehydrogenase (mLDH-A) HRE, human erythropoietin (EPO) HRE, and human VEGF HRE, have been examined for the development of artificial HIF-1-responsive promoters [118-124]. The number of HREs, interval between the HREs, and combination with the basal promoter influence the HIF-1-responsiveness of each HRE-containing promoter. Among candidates, the combination of five repeats of HRE derived from the human VEGF promoter and the human cytomegalovirus (CMV) minimal promoter (mp), the so-called “5HRE promoter”, showed intense HIF-1-responsiveness under hypoxic conditions. It exhibited a more than 500-fold increase in luciferase activity in response to hypoxic stimuli [124].

The 5HRE promoter was further modified to increase the HIF-1-dependency, because it shows a certain level of unwanted gene expression even under normoxic conditions [121]. In order to decrease the leakage under normoxic conditions, we fused the coding sequence of the HIF-1α ODD domain to that of luciferase in frame, and inserted it downstream of the 5HRE promoter. The resultant *5HREp-ODD-luc* reporter gene showed little leakage under normoxic conditions, which contributes to an increase in the hypoxia-responsiveness by up to 4.7 × 10. Also, addition of ODD realized the real-time degradation of ODD-Luc proteins in response to reoxygenation under the same destabilizing regulation as HIF-1α protein.

#### 4.2.2. Imaging of HIF-1 Activity in Tumor Xenografts

HIF-1-activity in tumor xenografts has been extensively analyzed using the HIF-1-dependent reporter genes. A human melanoma cell line, Be11, was stably transfected with *5HREp-d2EGFP*, which expresses a derivative of EGFP, d2EGFP, under the control of the 5HRE promoter. Immunodeficient nude mice were transplanted with the cells and subjected to an optical imaging experiment [125]. Resultant tumor xenografts showed heterogeneous and partition-dependent green fluorescence. Immunohistochemical analyses confirmed that d2EGFP-positive cells were located at the boundary between well-oxygenated viable regions and necrotic regions, which were also stained with a hypoxia marker, pimonidazole [114]. When a human cervical cancer cell line, HeLa which was stably transfected with the *5HREp-luc* or *5HREp-ODD-luc* gene was transplanted into nude mice, the resultant xenografts showed a certain bioluminescence. The bioluminescent intensity dramatically increased after the tumor-bearing leg was ligated and the blood flow to the xenograft decreased [22,121]. We have successfully obtained detailed information about the dynamics and changes of intratumoral HIF-1-activity during tumor progression and after radiation therapy.

Although the optical imaging strategies have contributed to basic research about tumor hypoxia, there is a limit of its usage in clinical applications. For example, we cannot exploit it for the evaluation of HIF-1activity in human cancers without an effective gene delivery system which enables us to deliver the reporter gene evenly to all the cancer cells composing the tumor. Moreover, because the strategy is fully dependent on the bioluminescent/fluorescent gene expression, which takes time in general, it is not good at detecting acute hypoxia. In addition, optical imaging has other disadvantages, such as poor spatial resolution and poor permeability and so on.

#### 4.2.3. Nuclear Medicine Imaging Based on HIF-1-Dependent Reporter Gene

Not only optical imaging but also several nuclear medicine imaging methods based on HIF-1-dependent reporter gene systems have been developed. The visualization of HIF-1 activation in rat glioma cells was accomplished by using the human sodium iodide symporter gene (hNIS) as a nuclear medicine reporter gene [126]. The radioactivity of ^99m^Tc, whose uptake is dependent on the expression of hNIS in tumors, was observed *in vivo* and the localization of accumulated radioactivity was similar to that of pimonidazole based on results of autoradiography and immunohistochemistry. He *et al.* reported the usefulness of the herpes simplex virus type 1 thymidine kinase (HSV1-TK) as another nuclear medicine imaging reporter gene combined with multiple copies of HREs for the imaging of HIF-1 activity [127]. They demonstrated that the intratumoral distribution of ^124^I-FIAU and ^18^F-FMISO was similar in human colorectal cancer cells. Although, nuclear medicine imaging systems can be applicable in humans, these kinds of reporter gene system still need the development of gene delivery methods for human application.

## 5. Conclusions and Perspectives

Based on the accumulated evidence described in the Section 2, it is obvious that both absolute hypoxic tumor cells and HIF-1 active cells are excellent targets to decrease the incidence of local tumor recurrence and distant tumor metastasis after radiation therapy. Hypoxia-selective cytotoxic drugs and HIF-1 inhibitors have been designed and used to overcome the problems. Hypo-IGRT has also been developed to deliver a booster dose of radiation to radioresistant fractions. In order to realize these strategies, it is critical to monitor the changes in the intratumoral localization and volume of both absolute hypoxic tumor cells and HIF-1 active cells.

Here, we introduced a number of potential strategies for assessing hypoxia and HIF-1 activity in malignant solid tumors; polarographic needle electrode, MRI, PET, optical imaging, immunohistochemical staining and so on. These methods promise a wide range of applications, not only for just detecting tumor hypoxia, but also for characterizing its biological features in support of personalized medicine for more effective cancer treatments. However, each method has its own weak as well as strong points. Optical imaging of intratumoral HIF-1 activity, polarographic needle electrode, and immunohistochemical staining using intrinsic and extrinsic hypoxia markers have greatly contributed to understanding basic biological characteristics of hypoxia in radiation oncology. However, all of them have disadvantages for clinical applications as described in Sections 3.1, 4.1, and 4.2.4. On the other hand, imaging strategies with MR and PET seems to be much more attractive because of their strong points, such as low invasiveness, high sensitivity, reproducibility, and repeatability. Especially, PET imaging has a great advantage that we can intentionally obtain not only morphological but also physiological and pathological features of hypoxia by choosing a suitable imaging probe for the purposes. Although the exposure to radiation has been sometimes pointed out as a disadvantage of PET imaging, the radiation dose is quite low and does not cause carcinogenesis; radiation dose for PET-CT and conventional fractionated radiation therapy is about 25 mSv and 60 Sv (1Gy = 1 Sv in case of X-ray) in average, respectively. In such a situation, researchers and physicians will inevitably face difficult questions; how can we choose an appropriate method to evaluate intratumoral hypoxia/HIF-1 activity? which technique is the best? and which is applicable in clinical activities? Thus, we have to unify a standard for assessing these diverse techniques for each purpose in clinical activities.

Intratumoral localization of both hypoxic regions and HIF-1-active cells changes during tumor growth and after radiation therapy more dramatically than we assumed. One important question to be answered from the clinical point of view is whether the timescale of the dynamics is identical to that in real human tumors. For this reason, it is necessary to analyze the dynamics of hypoxia and HIF-1-active cells in human cancers. Then, we can optimize the timing and frequency of hypoxia/HIF-1 imaging for the planning of both Hypo-IGRT and chemoradiotherapy with hypoxia/HIF-1-targeted drugs, leading to the realization of highly personalized and multidisciplinary radiation therapy.

## Figures and Tables

**Table 1.** Methods of assessing tumor hypoxia (low oxygen concentration) in malignant solid tumors.

**Strategies**	**References**
Polarographic needle electrode	[28,36]
BOLD-MRI	[76–79]
DCE-MRI	[80–84]
19F-MRI	[85,86]
EPR	[87,88]
PET	[89–112]

**Table 2.** Strategies to assess HIF-1 activity in malignant solid tumors.

**Strategies**	**Imaging Targets/Imaging Tools**	**References**
Immunohistochemistry	Extrinsic Markers: Pimonidazole, EF5,	[113,114]
	Intrinsic Markers: HIF-1α, VEGF, GLUT-1, CAIX	[115-117]
Optical Imaging	5HREp-luc/5HREp-ODD-luc Reporter Genes	[22,121]
Using HIF-1-dependent	5HREp-d2EGFP/5HREp-EGFP Reporter Gene	[125]
Reporter Gene	5HREp-DsRed2 Reporter Gene	[23]
Nuclear Medicine Imaging	5HREp-hNIS Reporter Gene	[126]
Using HIF-1-dependent Reporter Gene	9HREp-HSV1-Tk	[127]
